# Development of a shear force measurement dummy for seat comfort

**DOI:** 10.1371/journal.pone.0187918

**Published:** 2017-11-29

**Authors:** Seong Guk Kim, Chang-Yong Ko, Dong Hyun Kim, Ye Eun Song, Tae Uk Kang, Sungwoo Ahn, Dohyung Lim, Han Sung Kim

**Affiliations:** 1 Department of Biomedical Engineering and Research Institute for Medical Instruments & Rehabilitation Engineering, Yonsei University, Wonju, Gangwon, Republic of Korea; 2 Korea Orthopedics & Rehabilitation Engineering Center, Incheon, Republic of Korea; 3 Body & trim development team, Hyundai Motor Group, Uiwang, Republic of Korea; 4 Cozy international Co., Ltd, Ansan, Republic of Korea; 5 Department of Mechanical Engineering, Faculty of Mechanical and Aerospace Engineering, College of Engineering, Sejong University, Seoul, Republic of Korea; Beihang University, CHINA

## Abstract

Seat comfort is one of the main factors that consumers consider when purchasing a car. In this study, we develop a dummy with a shear-force sensor to evaluate seat comfort. The sensor has dimensions of 25 mm × 25 mm × 26 mm and is made of S45C. Electroless nickel plating is employed to coat its surface in order to prevent corrosion and oxidation. The proposed sensor is validated using a qualified load cell and shows high accuracy and precision (measurement range: −30–30 N; sensitivity: 0.1 N; linear relationship: R = 0.999; transverse sensitivity: <1%). The dummy is manufactured in compliance with the SAE standards (SAE J826) and incorporates shear sensors into its design. We measure the shear force under four driving conditions and at five different speeds using a sedan; results showed that the shear force increases with speed under all driving conditions. In the case of acceleration and deceleration, shear force significantly changes in the lower body of the dummy. During right and left turns, it significantly changes in the upper body of the dummy.

## Introduction

Seat comfort is of substantial interest to automotive engineers as it is one of the major factors that consumers consider when purchasing a car. Seat comfort has been evaluated using various methods. Thus far, the most common method for evaluating seat comfort has been questionnaires [1–3]. However, it is a qualitative evaluation method. Alternatively, quantitative evaluation methods have been suggested and used. Vibration, in particular, has been widely evaluated [4–6], but it may be inadequate for evaluating the seat comfort of drivers or passengers during driving because vibration is generated by several factors, including road conditions, car conditions, and seating. In addition, the interfacial pressure between the seat and the human body has been used to quantitatively evaluate seat comfort [7–10]. However, in some previous studies, no or less correlation between pressure and seat comfort has been shown [9, 10]. The conflicting results indicate that the interfacial pressure may be inadequate for evaluating the seat comfort of drivers or passengers.

Shear force or vibration in the plane of the car is considered as a determinant of seat comfort [11–13]. Some studies have developed and used shear sensors to measure the interfacial shear force between humans and machines [12, 13]. Cho et al. developed a shear-measurement sensor for measuring forces at the human–seat interface [12] and determined the range of the shear force between a human and a seat. The shear force was found to change according to human posture because the sensor was situated between the human and the seat [12]; this led to results with limited reproducibility. Alexandra et al. (2016) proposed and characterized the principle behind a novel compliant shear-force sensor comprising conductive and nonconductive silicone elastomers [14]. However, this sensor’s measurement range was limited to 2.6–6.8 N, which is insufficient to measure the shear force generated in a vehicle. Jonathan et al. evaluated 21 commercial wheelchair seat cushions using a commercial shear-force sensor [13]. However, as only one such sensor was used, it was difficult to simultaneously measure the shear force at multiple points.

When a shear-force sensor is placed between a human and the seat, the measurement of shear force is affected by the location of the sensor as well as the human’s posture and anthropometric or physical characteristics, e.g., weight. Moreover, emotional responses to external stimuli are dependent on body locations [15, 16]. Therefore, the development of dummy-type sensors is required. This study aims to develop a dummy incorporating several shear-force sensors in order to evaluate seat comfort.

## Materials and methods

### Fabrication of the shear sensor

**Fig 1:**
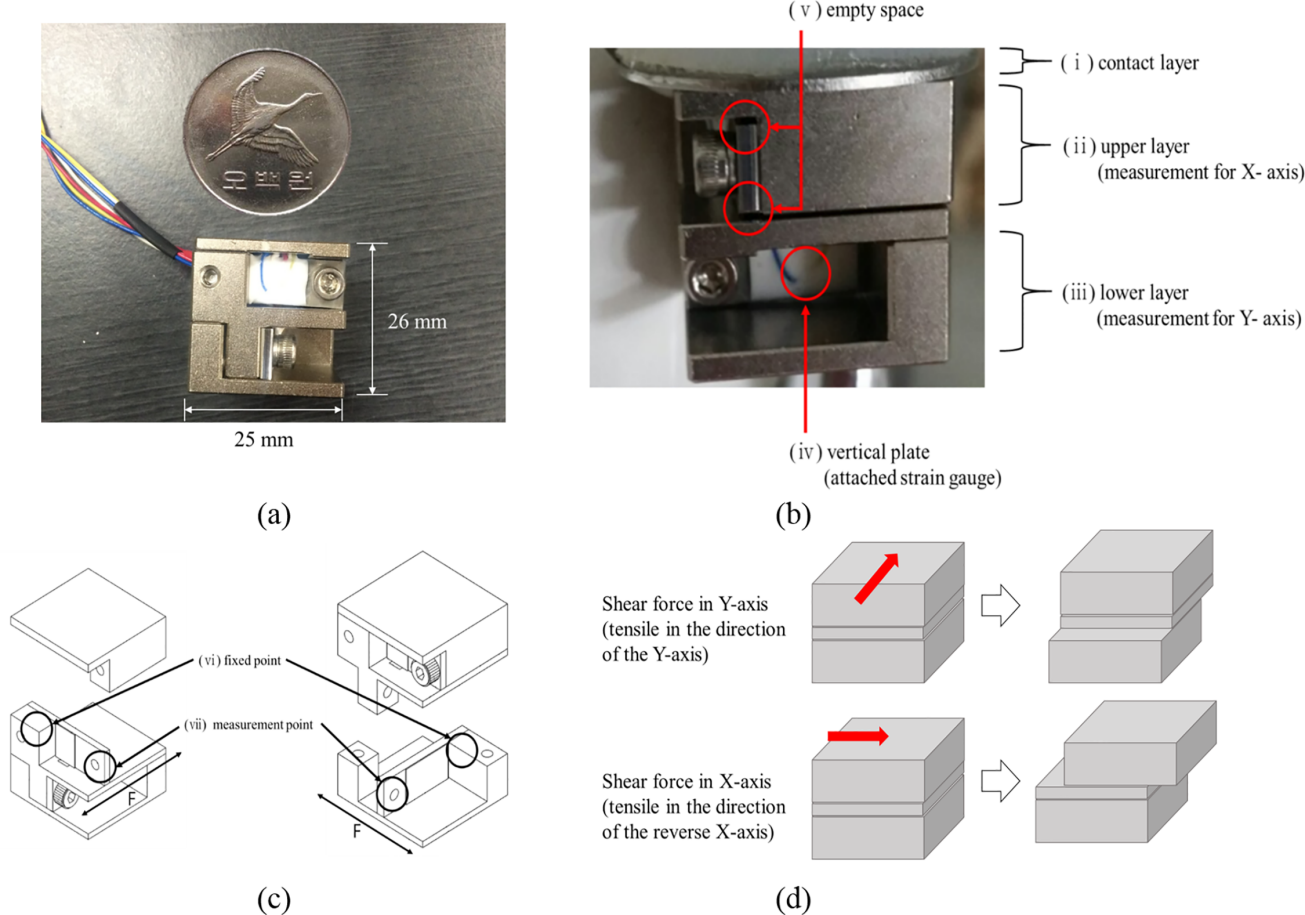
Views of shear sensor. (a) Front view, (b) side view, (c) inside view, and (d) shear-force measurement principle.

The fabricated sensor was designed as a hexahedron so that it could measure shear forces in any direction. Its dimensions (width × length × height: 25 mm × 25 mm × 26 mm, shown in Fig 1A) were determined according to the structure of the sensor and the size of the strain gauge. The sensor was made of S45C; its surface was plated via the electroless nickel plating technique to prevent corrosion and oxidation. S45C Steel Grade is a steel grade material, which is standard specification of carbon steels for machine structural use. S45C was used as a material for parts requiring mechanical strength and was widely used for various gears, shafts, chains, rollers, molds, and pins. Teflon tape with a small friction coefficient was used to reduce the frictional force inside the sensor.

The sensor comprised three layers (contact, upper, and lower, with heights of 2, 12, and 12 mm, respectively; Fig 1B). The contact layer transmitted shear forces to the seat. The upper layer measured the shear force along the X axis, and the lower layer measured the shear force along the Y axis (Fig 1C). The upper and lower layers were fixed to vertical plates. These plates, which directly measured the shear force, were made using a load cell (CZ2A, COZY INTERNATIONAL, KOREA). Space was provided between each layer fixed to the vertical plates in order to prevent or minimize the interference of the normal force.

### Validation of sensors

**Fig 2:**
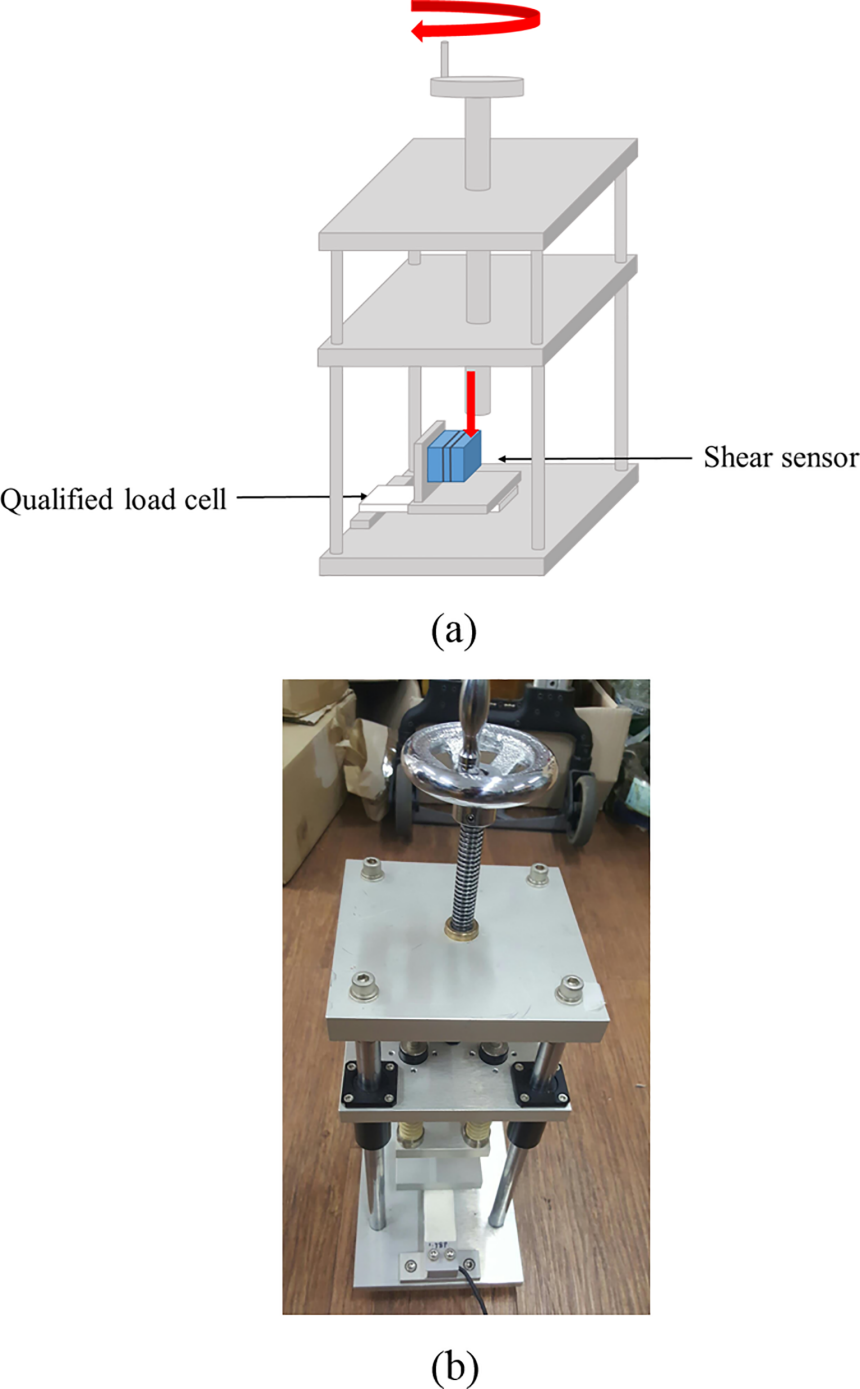
Calibration device. (a) Calibration principle for shear sensor and (b) photo of the calibration device.

The shear-force measurement principle is shown in Fig 1D. Briefly, when a shear force was applied to the contact layer of the sensor, deformation of the strain gauge in the vertical plate generated electronic signals. Shear force was calculated using the strain gauge of the shear sensor and linearized using Eq (1) as follows:

Fτ=Kτ×Eτ (1)

where Fτ is the shear force, Kτ is a constant, and Eτ is the change in the strain value of the strain gauge. To validate this equation, this study used a calibration device with a qualified load cell (CZ1P, COZY INTERNATIONAL, KOREA; Fig 2). The target range for the measurement of shear force at the human–seat interface was set to −30–30 N based on previous findings [12]. Calibration was performed as follows. First, the shear sensor was placed in the calibration device. The knob on the calibration device was turned, and the shear sensor and the qualified load cell attached to the calibration device were simultaneously loaded. The load was converted to newtons (N) using the qualified load cell. When the load cell was in the range of −30–30 N, the value measured by the shear sensor was checked and Kτ was adjusted to generate a load of −30–30 N. After the calibration of the X axis, the other axis was calibrated using the same method. A total of 30 shear sensors were fabricated. To achieve high accuracy, Kτ was calculated for all sensors. In this study, a data acquisition board was manufactured for signal measurement and calibrated using CB-2R (TOKYO SOKKI KENKYUJO CO., LTD., JAPAN). To gauge transverse sensitivity, when the force was measured in one direction, the influence in the other direction was evaluated. A linear-regression analysis was performed to evaluate the correlation between the applied force and the force measured by the shear sensor. Bland–Altman analysis was then performed to verify the accuracy and reliability of the shear sensor. To validate the sensor, the characteristics of the sensor were measured at least three times.

### Development of the dummy

**Fig 3:**
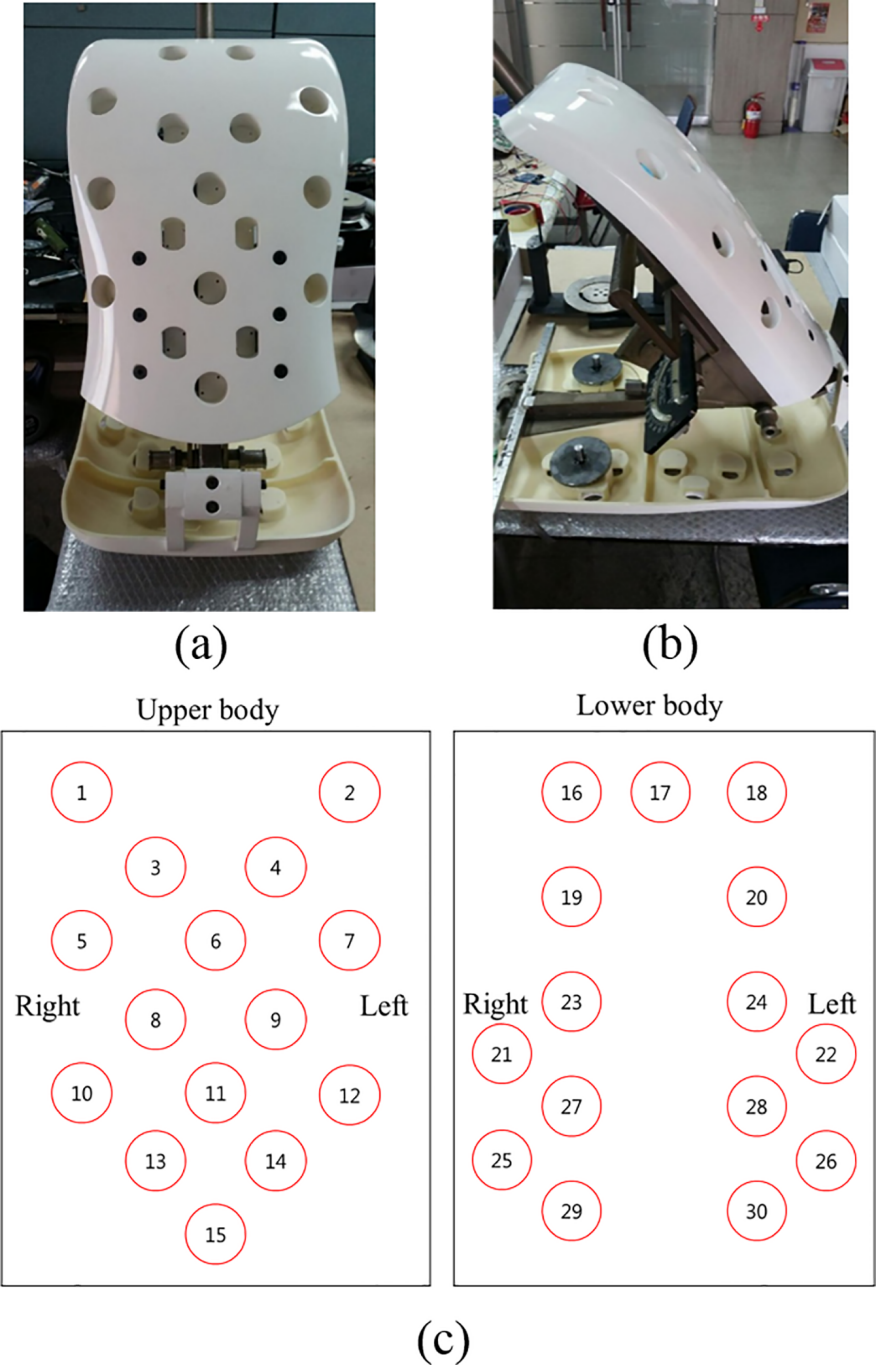
Developed dummy. (a) Front view, (b) side view, and (c) mounting position of the sensors.

The dummy was made in compliance with the Society of Automotive Engineers (SAE) standards (Fig 3A and 3B) [17]. The SAE standards were used to obtain the dimensions of the passenger compartment or evaluate vehicle seats. The dummy was set to 50th percentile (weight: 77 kg, torso length: 563 mm, thigh length: 431.5 mm, material: acrylonitrile butadiene styrene, and thickness: 1–1.5 cm) in accordance with the SAE dummy. The weight distribution was set to the same weight position of SAE dummy using the SAE dummy frame. A total of 30 sensors were attached to the developed dummy: 15 to the upper body and 15 to the lower body (Fig 3C). The mounting positions were selected along the curved surfaces and areas where the bodyweight is typically highly concentrated. In the upper body, several sensors were attached to the spine. In the lower body, several sensors were attached to the thighs to enable the measurement of shear force applied on the side bolster in the seat. The contact layer of the shear sensors was attached to the silicon plate to prevent slipping (thickness: 1.5 mm). When the shear sensors were attached to the dummy, the silicon plate and contact layer protruded from the surface of the dummy (1–2 mm).

### Pilot study using the shear-force measurement dummy

**Fig 4:**
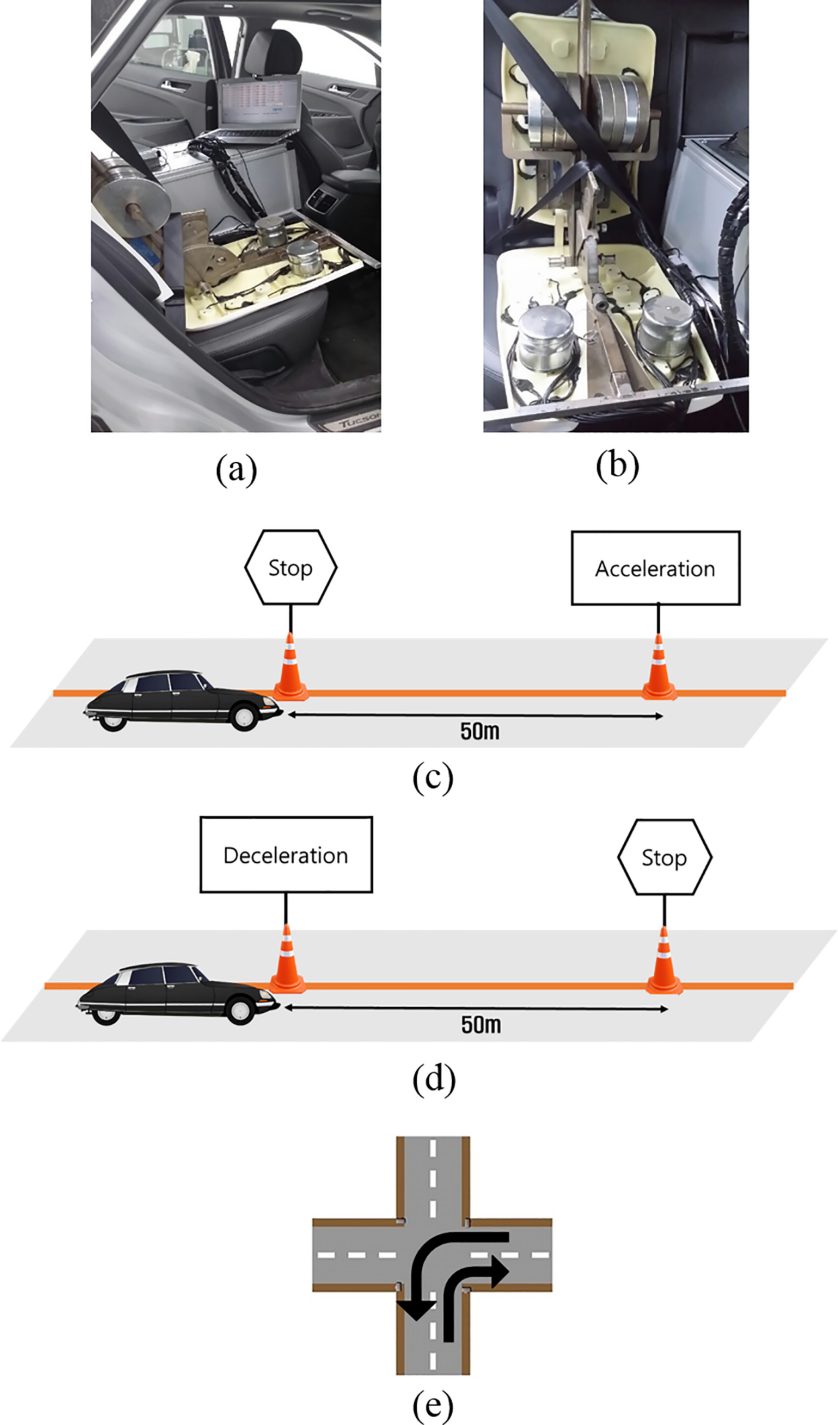
Experiment setup and courses of four driving conditions. (a) Side view of the test position, (b) front view of the test position, (c) acceleration, (d) deceleration, and (e) right turn and left turn.

This study used a large sedan as the vehicle test. The test position was determined to be behind the passenger seat in the vehicle (Fig 4A and 4B). The angle of the backrest and seat was adjusted to be neutral (54.2° and 16.6°, respectively). Four driving conditions were used in this experiment, as shown in Table 1 and Fig 4C–4E. For acceleration testing, the car started at a speed of 0 km/h and reached one of five speeds as it passed the 50-m mark. For deceleration, the car started at each of the five speeds and reached 0 km/h at the 50-m mark. Right and left turning were tested under the same conditions on a two-lane road at each of the specified speeds. Three trials were conducted for each test. Throughout the experiment, a skilled professional driver drove the car.

**Table 1:** Driving conditions.

Driving conditions	Speed	Method
Acceleration	30, 40, 50, 60, and 70 km	Acceleration for 50 m with five speeds from the stop state
Deceleration	30, 40, 50, 60, and 70 km	Deceleration for 50 m with five speeds from the driving state
Right turn	10, 15, 20, 25, and 30 km	Right turn at the corner with five speeds
Left turn	10, 15, 20, 25, and 30 km	Left turn at the corner with five speeds

## Results

### Results of sensor validation

**Fig 5:**
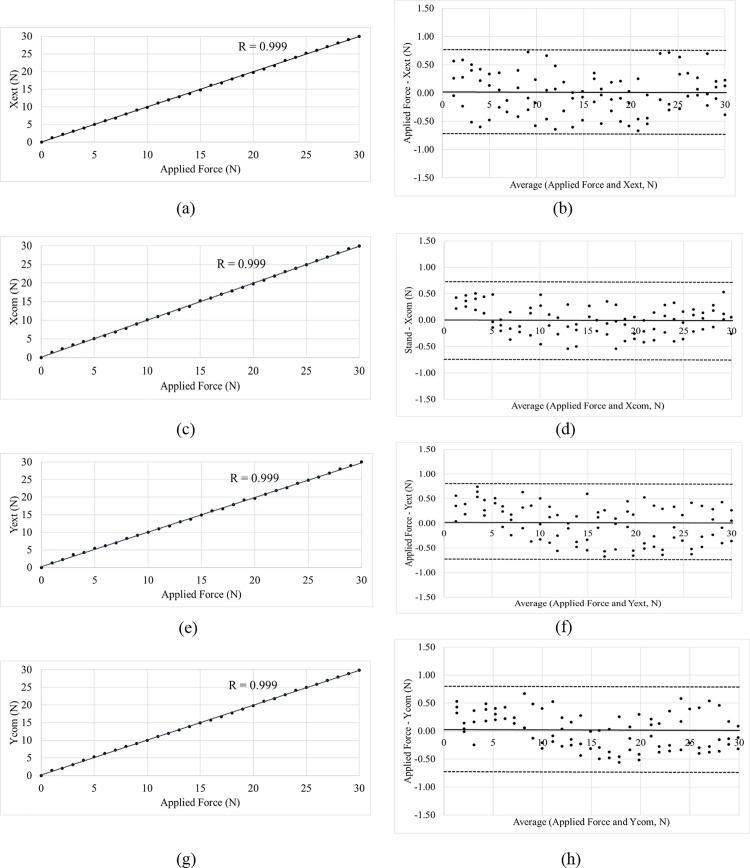
Validation result of the shear sensor. (a) Correlation test at Xext, (b) Bland–Altman at Xext, (c) correlation test at Xcom, (d) Bland–Altman at Xcom, (e) correlation test at Yext, (f) Bland–Altman at Yext, (g) correlation test at Ycom, (h) Bland–Altman at Ycom, solid line: mean difference; dashed line: mean ± 1.96 standard deviations; Xext, tensile in the direction of the X axis; Xcomp, tensile in the direction of the reverse X axis; Yext, tensile in the direction of the Y axis; Ycomp, tensile in the direction of the reverse Y axis.

The sensitivity of the sensors was 0.1 N; the transverse sensitivity was found to be less than 1% in all sensors. The linear relationship was found to be close to 0.999. Furthermore, the accuracy and precision were confirmed via the Bland–Altman analysis (Fig 5). The mean of the differences was close to 0 (between −0.009 and 0.013 N), and the differences were all located within ±1.96 standard deviations. Based on the results of sensor validation through at least three repetitive measurements, it was concluded that the sensor is sufficient to measure the shear force when used in the dummy.

### Results of the pilot study

**Fig 6:**
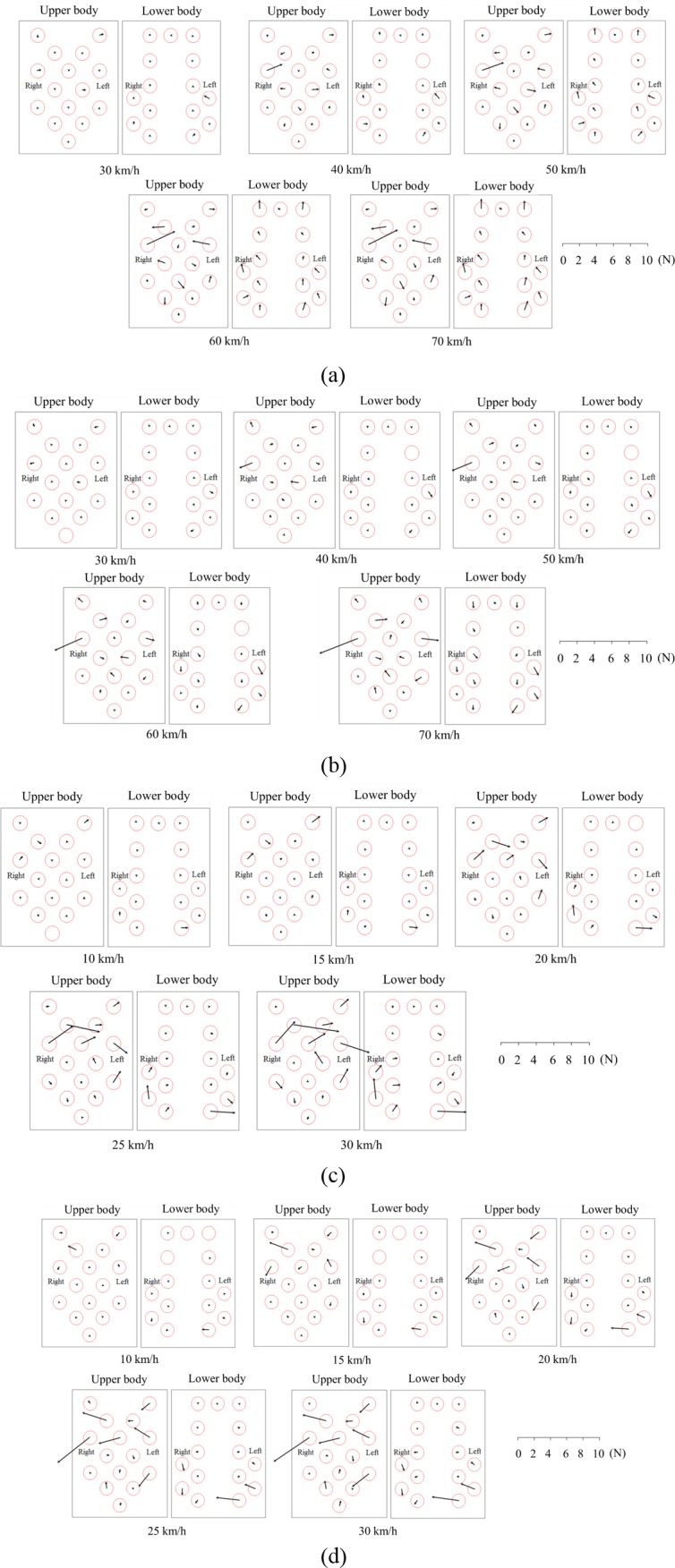
Results of the vehicle test. (a) Acceleration, (b) deceleration, (c) right turn, and (d) left turn.

The results of the vehicle test are shown in Fig 6. It was confirmed that the shear force increased with speed under all driving conditions. Shear forces in the upper body were found to be higher than those in the lower body under all driving conditions.

While driving along a straight path, the changes in the shear force on the upper part of the upper body and the buttocks were larger than those on other body parts. Shear forces were generated toward the center of the upper body in the case of acceleration, whereas they were generated outward from the center of the upper body in the case of deceleration.

While turning, the changes in shear force on the upper part of the upper body and the distal end of the thigh were larger than those on other body parts. When taking a right turn, the change in the shear force was larger toward the left, whereas when taking a left turn, it was larger toward the right.

## Discussion

In this study, shear sensors were developed and validated. A dummy incorporating shear sensors was developed and used to measure the shear force on car seats during driving.

The Bland–Altman plot and correlation results indicate a good agreement between the shear sensor and the load cell. Additionally, an even distribution of differences between the shear sensor and the load cell indicate a low systematic bias [18]. Transverse sensitivity and cross-talk were less than 1%. These results indicate that the shear sensor was validated in the range of −30–30 N.

The resultant shear force increased with acceleration, regardless of the driving condition; this might be attributed to inertia. The direction of the shear force depended on the driving condition. The results show that the upper body moved down while the lower body moved in the direction opposite to that of the vehicle during acceleration. The reasons for this result are as follows. The lower body moved in a direction opposite to that of the vehicle owing to inertia and downward owing to the seat angle and body weight. As a result, the lower body moved downward such that the upper body connected to the lower body also moved downward. The upper body moved up and the lower body moved in the vehicle’s direction of travel during deceleration. The reasons for this result are as follows. The lower body moved in the direction of travel owing to inertia and moved upward owing to the seat angle. As a result, the lower body moved upward such that the upper body also moved upward. The upper and lower bodies moved toward the left when the vehicle turned right and toward the right when the vehicle turned left. This result was also due to inertia.

Interestingly, the right shear force on the upper body seemed to be higher than the left one during driving along a straight path even though there was an even weight distribution between the left and right sides. If the dummy was not fastened by a seat belt, the right side of the upper body during left turning or the left side of the upper body during right turning would have moved differently compared with the case when the seat belt was fastened; this would lead to undetectable shear force on the right or left sides. Such forces were shown in this study. Here, the dummy was fastened using a three-point retractable seat belt. To the best of our knowledge, this seat belt had no direct effects on the shear force. However, some studies have shown that injuries on the right side of the body are more serious than those on the left side [19, 20]. These results may imply that the retraction force can generate high tension, leading to high compression on the right side. Therefore, the differences in the shear force between the right and left sides might be due to the influence of the seat belt. As a result, a combination of inertia and retraction force might have generated on the right upper body. Therefore, the influence of seat belt on shear force should be considered in future research.

This study has several drawbacks. First of all, the influence of seat belts was not considered. Moreover, human anatomical features, such as the anatomy of hard tissues or the biomechanics of soft tissues, were not considered for the dummy, and only one seat posture and dummy position was evaluated. To obtain reliable results, several postures and dummy positions should be considered. Moreover, the results were affected by human errors. Human errors occur when the person judges the speed on the scale of the instrument panel and steps on the accelerator or brake pedal. The average error in the shear-force measurement dummy was 14.7% after three repetitive measurement experiments. This error should be considered when analyzing the experimental results because the vehicle is driven manually. Moreover, the movement of the passenger in the car seat is limited. Therefore, to study seat comfort, movement in the car seat must be considered [21]. Comfortable seats should accommodate postural changes [22] and changes in the body position should be allowed to relax and relieve the muscles [23]. When the passenger is sitting on the seat for a long time, the passenger moves the body and releases the muscles. These behaviors have individual differences, and as the duration of sitting increases, the sense of discomfort in the passenger also increases. Therefore, the final goal is to develop a seat that is comfortable for the passenger even after sitting for a long time. For this, it is necessary to consider the seat material, spring, cushion, shape, and other factors. In addition, it is necessary to evaluate how physical effects such as pressure, shear force, and vibration affect the passenger. Therefore, many studies have developed equipment to measure these effects. In this study, a shear-force measurement dummy was developed and evaluated to measure the shear force among physical influences.

## Conclusions

In conclusion, we developed a dummy for measuring the shear force on car seats during driving. The dummy comprised several shear sensors that were validated and proved to be effective. The shear sensor was designed to measure shear force in the range of −30–30 N. Throughout the experiments, there was no saturation of shear-force sensors. Therefore, the range seemed to be reasonable for measuring the shear force on car or vehicle seats during driving. In the further study, these results are then used to determine the correlation between subjective comfort and shear force.
